# A Probabilistic Model of Fatigue Life at the Interface of CRTS II Slab Ballastless Track

**DOI:** 10.3390/ma19132762

**Published:** 2026-06-29

**Authors:** Anxiang Song, Yuanchen Guo, Guowen Yao, Xuanrui Yu

**Affiliations:** 1 College of Materials Science and Engineering, Chongqing University, Chongqing 400044, China; 2 School of Civil Engineering, Chongqing Sanxia University of Science and Technology, Wanzhou, Chongqing 404100, China; 3 School of Civil Engineering, Chongqing Jiaotong University, Chongqing 400074, China; yaoguowen@sina.com; 4 School of Civil and Hydraulic Engineering, Chongqing University of Science & Technology, Chongqing 400074, China; 2024007@cqust.edu.cn

**Keywords:** CRTS II slab ballastless track, Weibull distribution, interfacial fatigue, probabilistic model

## Abstract

The gradual deterioration of interfacial performance in CRTS II (China Railway Track System II) slab ballastless tracks during long-term service can significantly affect structural stability and durability. Existing studies have mainly focused on the fatigue performance of the overall track system and individual structural layers, whereas probabilistic fatigue-life modeling of the interlayer interface remains relatively limited. This study investigates the fatigue life behavior of the track slab-CA (cement-asphalt) mortar interface under cyclic loading. An exponential stress life relationship was combined with a two-parameter Weibull distribution of fatigue life at a specified stress ratio to establish a multi-parameter Weibull-based probabilistic framework that links fatigue life, stress ratio, and failure probability. Push-out and positive tensile fatigue tests were conducted on composite specimens to obtain interface fatigue lives under different stress ratios. Leveraging the multi-parameter Weibull model and experimental data, the L-BFGS-B (Limited-memory Broyden-Fletcher-Goldfarb-Shanno with Box constraints) algorithm was employed to optimize the model parameters and construct a probabilistic fatigue life model. The calibrated model was then used to analyze the fatigue behavior of the slab-CA mortar interface under tangential and vertical loading. The results show that the proposed probabilistic framework provides good agreement with the interface fatigue test data and enables the fatigue-life distribution and failure probability of the interlayer interface to be evaluated under different stress ratios. The findings provide a probabilistic basis for fatigue assessment and durability analysis of CRTS II slab ballastless track interfaces.

## 1. Introduction

**Figure 1:**
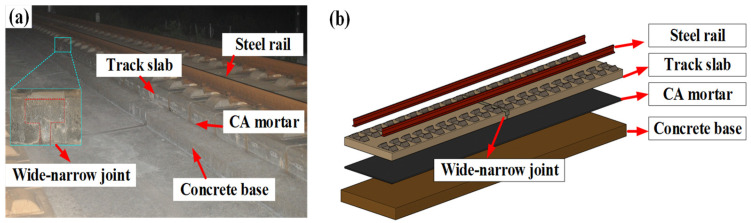
CRTS II slab Ballastless track: (**a**) On-site picture; (**b**) Structure schematic diagram.

Slab ballastless track is the predominant structural form in modern track construction, extensively utilized in high-speed railroads [1,2,3] and urban rail transit systems, such as subways [4,5,6,7]. The CRTS II slab ballastless track, developed through technology introduction and re-innovation, is extensively deployed in high-speed railroad lines such as Beijing-Shanghai, Shanghai-Kunming, Beijing-Tianjin, and Shanghai-Hangzhou, owing to its high geometric precision, structural integration, and longitudinal continuity [8]. The total operational mileage of this system now exceeds 8000 km [9]. This system primarily comprises a multilayer heterogeneous structure, including precast track slabs, CA mortar, and concrete base, with adjacent track slabs connected by T-joints to form a longitudinally continuous track structure, as illustrated in Figure 1.

**Figure 2:**
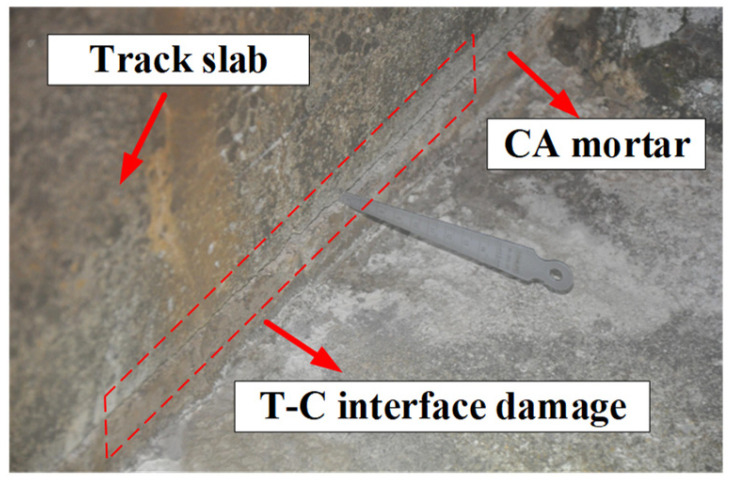
CRTS II slab ballastless track T-C interface damage disease.

However, as a multi-layer composite structure, the CRTS II slab ballastless track features heterogeneous interfaces, which are among the most vulnerable elements within the track structure system. Field investigations have revealed that the CRTS II slab ballastless track is susceptible to interface damage between the track slab and CA mortar layer (referred to as the T-C interface) under external train loads, with interface performance progressively deteriorating over prolonged service, as depicted in Figure 2. Furthermore, studies have identified interface damage as a primary contributor to other structural issues, significantly compromising the overall stability of the system. Thus, evaluating the interface fatigue life of track structures is crucial for ensuring their safety and reliability in service.

Currently, extensive research has been conducted on the fatigue life of track structures, focusing primarily on the performance of structural layers and interlayers. Regarding the structural layers, numerous studies have been conducted on the fatigue performance of various types of track structures. Yang et al. [10] conducted fatigue cyclic loading tests on CRTS II slab ballastless track panels, demonstrating that fatigue load levels significantly affect the damage to track slabs under variable amplitude loading. Terifa et al. [11] introduced a full-size three-point bending fatigue test and established a mapping relationship between failure critical displacement, displacement rate, and fatigue life, enabling accurate fatigue life prediction. In addition, Poveda et al. [12] developed a finite element model of a three-slab track system to numerically investigate the fatigue life design of concrete slabs. Khajehdezfuly et al. [13] created a numerical model of vehicle/slab track interaction and demonstrated that the fatigue life of concrete slabs can be enhanced by decreasing rail pad stiffness and increasing compartment stiffness through parametric analysis. Zeng et al. [14,15] investigated fatigue damage in CRTS III slab ballastless track under heavy train loading, highlighting the fatigue characteristics and cracking of the base concrete slab. Xu et al. [16] developed a fatigue damage model to evaluate the fatigue performance of self-compacting concrete (SCC) in CRTS III slab track under varying stress levels, revealing that SCC fatigue life decreases as maximum stress levels increase. The results show that the fatigue life of SCC decreases with the increase in the maximum stress level. The strain evolution and elastic modulus degradation were categorized into three stages: “fast-slow-fast”. Additionally, cement mortar (CM) specimens were tested at four maximum stress levels to assess the flexural fatigue performance of CM in unprotected track concrete and to develop a corresponding fatigue damage model. Results indicated that higher fatigue loads caused more significant damage in the early stages and less damage in the later stages [17]. Deng et al. [18] integrated the high peripheral fatigue damage ontological relationship of CA mortar into a refined finite element model of CRTS I ballastless track and established a fully coupled damage finite element method, demonstrating that the influence of pore space on fatigue damage accumulation in CA mortar exceeds that of initial deterioration and wheel load changes. Chen et al. [19] developed a mesoscale thermal-force coupled finite element model for concrete slab tracks, incorporating a nonlinear damage accumulation method based on stress-time history to estimate the fatigue life of CA mortar under combined thermal and vehicle loading. Their findings indicated that thermal effects significantly reduce the fatigue life of CA mortar, with considerable variations observed across different locations.

In addition, the performance of the interlayer interface has been extensively studied through theoretical analysis, laboratory testing, and numerical simulations. Xia et al. [20] integrated the meshless radial point interpolation method with higher-order shear deformation theory (HSDT) to develop an interlayer slip model. They compared this model with the double-layer plate model to verify and analyze the influence of boundary conditions and elastic foundation coefficients on the structural stresses of the interlayer. Also, scholars and research institutes have conducted full-scale modeling tests to assess the shear-bearing capacity of the interface between the track slab and the CA mortar layer, deriving the corresponding load–displacement relationships [21,22]. Song et al. [2], Xu et al. [23], and Du et al. [24] conducted splitting tensile and push-out tests primarily on small specimens, employing digital image correlation (DIC) to measure and calibrate the parameters of the interface deformation field. Sun et al. [25] performed static and fatigue split tensile tests to determine the damage characteristics of the interface between the track slab and self-compacting concrete, revealing the evolution of damage variables relative to fatigue life ratios under different stress levels. Zhu et al. [26] conducted indoor tests of interlayer normal and tangential cracking on interlayer bonded structural members of double-block ballasted track, analyzed the interlayer damage cracking behavior and intrinsic parameters of the specimens, and obtained interface fatigue S-N curves. He et al. [27] conducted fatigue tests on C60-SCC composite specimens, obtained the interlaminar interfacial degradation law and the bond response of CTRS III slab ballastless track and predicted the interfacial fatigue life by combining them with the S-N curves. Wang et al. [28] proposed an improved fatigue bond zone model that accounts for time-varying factors and analyzed the fatigue performance evolution of the interlaminar track plate and SCC under varying bond strengths, uncovering the mechanism of fatigue performance evolution between the layers.

Numerous studies have been conducted on the fatigue performance of track structure systems, including different structural layers and interlayer interfaces, which have laid a solid foundation for exploring the fatigue damage evolution and predicting the fatigue life of track structures. It is important to note that under a given stress level, the loading amplitude in the interface fatigue test remains constant, but the fatigue life of the interface exhibits random variability. This paper employs probability functions to describe the stochastic nature of the fatigue life data. Currently, both the normal distribution and Weibull distribution functions are primarily used for this purpose [29]. The Weibull distribution model is highly regarded for its superior data-fitting capabilities and straightforward formulation, making it well-suited for modeling fatigue failure as service life progresses. This model is particularly effective in addressing challenges associated with fatigue behavior, especially in cases involving small sample sizes [30,31,32]. Castillo et al. [33,34] previously proposed a multiparameter Weibull distribution fatigue probability model for steel wire fatigue studies, highlighting the advantages and necessity of the Weibull distribution in describing fatigue life. Lan et al. [35] developed a Weibull model for the corrosion of steel reinforcement based on the S-N relationship, enabling the evaluation of reinforcement bar fatigue life under natural and artificially accelerated corrosion conditions. Huang et al. [36] calculated the fatigue life of sisal fiber foam concrete using the Glivenko-Cantelli theory and Weibull distribution function, demonstrating that three-parameter Weibull analysis yields more accurate results. Similarly, Wang et al. [37] employed a three-parameter Weibull distribution for reliability analysis and life prediction, addressing the stochastic nature of fatigue life in mechanical parts, and proposed an unconstrained optimization method for parameter estimation. Given the limited amount of test data, Liu et al. [38] and Wang et al. [39] utilized the Weibull distribution to process small sample sizes and established P-S-N curves under different failure probabilities to elucidate the relationship between stress and fatigue life. However, research on the fatigue performance of the CRTS II slab ballastless track interlayer remains limited. Furthermore, the lengthy duration and high cost of fatigue tests pose challenges in obtaining substantial test data for analysis. Additionally, given the fatigue resistance of the CRTS II slab ballastless track structure interface and the randomness of applied loads, the fatigue behavior of the track structure interface exhibits stochastic characteristics. Thus, selecting an appropriate model and accurately determining its parameters are critical for predicting the fatigue life of the track structure when processing limited test data.

To fill the above research gaps, this paper advances the development of a probabilistic fatigue life model for the CRTS II slab ballastless track interface, building upon existing research findings. Based on the exponential function stress-life model and the assumption that fatigue life under a specified stress ratio follows a two-parameter Weibull distribution, a multi-parameter Weibull model is derived to characterize fatigue life. Push-out and positive tensile fatigue tests are conducted, and the model parameters are numerically optimized using the L-BFGS-B algorithm, informed by the multi-parameter Weibull model and test results. A probabilistic fatigue life model is then established to analyze the fatigue life of push-out and positive tensile tests.

### Research Significance

Compared to the published findings of existing studies, this study provides a comprehensive investigation into the interfacial fatigue life probability model of the CRTS II slab ballastless track. While previous research has extensively examined the fatigue performance of track structure systems, including various structural layers and interlayer interfaces, the probabilistic fatigue life model of the interlayer interface has not been thoroughly explored. In this study, a fatigue life probabilistic model was developed by conducting positive tensile and push-out fatigue tests on composite specimens of the track slab, CA mortar, and concrete base. A multi-parameter Weibull model was derived to characterize the fatigue life, followed by numerical optimization. Additionally, the parameters for both tangential and normal fatigue life models were obtained using interface fatigue test results and parameter estimation through the maximum likelihood estimation and the L-BFGS-B algorithm. This further allowed for the analysis of the model’s applicability. The findings of this study hold significant practical value for fatigue calibration and safety assessment of the interlayer interface in CRTS II slab ballastless tracks.

## 2. Probabilistic Fatigue Modeling of Ballastless Track Interlayer Interfaces

### 2.1. Fatigue Life Probability Model

The S-N curve (stress-life model) for the T-C interface of CRTS II slab ballastless track can be expressed using the Basquin model, which can be described as:

(1) lgN=α+βσ

where *σ* is the stress ratio, *N* is the fatigue life at the stress ratio, *α* and *β* are material constants. Assuming that the parameters *α* and *β* of Equation (1) both depend on the probabilistic assurance rate *P*, for a given probabilistic assurance rate *P*, the P-S-N curve is obtained from Equation (1):

(2) $$\mathrm{lg}N_{P}=\alpha_{P}+\beta_{P}\sigma$$

where *N_P_* is the fatigue life for a given probability margin *P*, *α* and *β* are the model parameters for a given probability margin *P*.

For a specified stress ratio *σ*, the fatigue life *N* of the interface obeys a two-parameter Weibull distribution, as shown in Equation (3):

(3) $$F(N;\;\sigma )=1-\exp\left[-{\left(\frac{N}{\varphi (\sigma )}\right)}^{\chi }\right]$$

where *χ* is a shape function of the Weibull distribution, independent of *σ*. *φ*(*σ*) is a function of the specified stress ratio *σ*, which characterizes the characteristic parameter of the Weibull distribution at the specified stress ratio *σ*, i.e., the characteristic fatigue life of the interface at the specified stress ratio σ, which decreases with the increase in the stress ratio. A logarithmic transformation of both sides of Equation (3) results in the following.

(4) $$\log N=\frac{1}{\chi }\log\left[-\ln\left(1-F\right)\right]+\log\left[\varphi (\sigma )\right]$$

Taking the same probability assurance rate *P* = 1 − *F* as Equation (2), comparing Equation (2) and Equation (4), and according to the requirement of consistency condition, one reasonable expression for *φ*(*σ*) is as follows:

(5) $$\varphi (\sigma )=K\sigma^{-B}$$

Substituting Equation (5) into Equation (3), Equation (3) can be rewritten as

(6) $$F(N;\sigma )=1-\exp\left[-{\left(\frac{\sigma^{B}N}{K}\right)}^{\chi }\right]$$

Then Equation (6) can be used as a multi-parameter Weibull model to characterize the fatigue life model, where *B*, *χ*, and *K* are the pending parameters of the model, and the corresponding probability density function is as in Equation (7).

(7) $$f(N;\sigma )=\chi \frac{N^{\chi -1}}{{\left(K\sigma^{-B}\right)}^{\chi }}\exp\left[-{\left(\frac{N}{K\sigma^{-B}}\right)}^{\chi }\right]$$

The probability density function yields the mean and variance as shown in Equations (8) and (9), respectively.

(8) $$E\left(N;\sigma \right)=\left(K\sigma^{-B}\right)\Gamma \left(1+\frac{1}{\chi }\right)$$

(9) $$V\left(N;\sigma \right)={\left(K\sigma^{-B}\right)}^{2}\left[\Gamma \left(1+\frac{2}{\chi }\right)-{\left(\Gamma \left(1+\frac{1}{\chi }\right)\right)}^{2}\right]$$

From Equation (6), the fatigue life *N* obeys a two-parameter Weibull distribution with shape parameter *χ*, and characteristic parameter *Kσ^−B^* for a specified stress ratio σ. In addition, the S-N curve is shown in Equation (2) for a given probability guarantee rate *P*. When the fatigue life *N* is specified, the stress ratio σ also obeys a two-parameter Weibull distribution with shape parameter *Bχ* and characteristic parameter *(K/N)*^−1/*B*^. Furthermore, if *H = σ^B^N*, the intermediate variable *H* obeys a two-parameter Weibull distribution from Equation (6). It should be emphasized that the threshold parameter in the three-parameter Weibull formulation is interpreted here as a statistical location parameter rather than an absolute physical fatigue limit. This parameter describes the lower-bound shift in the stress-ratio distribution under a specified fatigue life within the tested data range. It does not imply that interface failure will never occur below this value. For the slab-CA mortar interface, inherent defects, material heterogeneity, viscoelastic behavior, and environmental degradation may still lead to damage accumulation under long-term cyclic loading. Therefore, the threshold parameter obtained in this study should be regarded as a specimen-scale statistical threshold under the tested loading conditions, rather than a universal endurance limit for field-scale CRTS II track structures.

(10) $$F(N;\sigma )=1-\exp\left[-{\left(\frac{H}{K}\right)}^{\chi }\right]$$

The fit of the model to the fatigue test data can be assessed using the Weibull probability plot of the intermediate variable *H*, provided that the model parameters are known. This analysis will be discussed in detail later in this paper.

### 2.2. Estimation of Fatigue Model Parameters

#### 2.2.1. Extreme Likelihood Method

The above model was derived under the condition of a specified stress ratio. From Equation (6), it can be seen that the model’s parameters can be estimated using fatigue data across various stress ratios. The fatigue test incorporates *t* stress ratios *σ_i_* (*i* = 1, 2, …, t), with *n_i_* fatigue test data *N_ij_* (j = 1, 2, …, *n_i_*) corresponding to each stress ratio. The unknown parameters in Equation (6) can be estimated using the maximum likelihood method, with the likelihood function expressed as follows:

(11) $$L\left(B,\chi ,K\right)=\prod_{i=1}^{k}\prod_{j=1}^{n_{i}}f\left(N_{ij};\sigma_{i}\right)$$

Substituting Equation (7) into Equation (11), and then Equation (11) becomes

(12) $$L\left(B,\chi ,K\right)=\prod_{i=1}^{k}\prod_{j=1}^{n_{i}}\chi \frac{N^{\chi -1}}{{\left(K\sigma^{-B}\right)}^{\chi }}\exp\left[-{\left(\frac{N}{K\sigma^{-B}}\right)}^{\chi }\right]$$

Further simplification of Equation (12) gives the likelihood function as shown in the following equation.

(13) $$L\left(B,\chi ,K\right)=\chi^{\sum_{i=1}^{k}n_{i}}{\prod_{i=1}^{k}\prod_{j=1}^{n_{i}}\left(\frac{N_{ij}}{K\sigma_{i}^{-B}}\right)}^{\chi -1}\frac{1}{K\sigma_{i}^{-B}}\exp\left[-\sum_{i=1}^{k}\sum_{j=1}^{n_{i}}{\left(\frac{N_{ij}}{K\sigma_{i}^{-B}}\right)}^{\chi }\right]$$

Therefore, the final expression of the likelihood function is shown in Equation (14).

(14) $$L\left(B,\chi ,K\right)=\chi^{\sum_{i=1}^{k}n_{i}}{\prod_{i=1}^{k}\prod_{j=1}^{n_{i}}\left(\frac{N_{ij}}{K\sigma_{i}^{-B}}\right)}^{\chi -1}\left(\prod_{i=1}^{k}\prod_{j=1}^{n_{i}}\frac{1}{K\sigma_{i}^{-B}}\right)\exp\left[-\sum_{i=1}^{k}\sum_{j=1}^{n_{i}}{\left(\frac{N_{ij}}{K\sigma_{i}^{-B}}\right)}^{\chi }\right]$$

Taking logarithms of both ends of Equation (14), then Equation (14) becomes

(15) $$\begin{array}{l} InL\left(B,\chi ,K\right)=\left(\sum_{i=1}^{k}n_{i}\right)In\chi +\left(\chi -1\right)\left(\sum_{i=1}^{k}\sum_{j=1}^{n_{i}}InN_{ij}-\sum_{i=1}^{k}n_{i}InK+B\sum_{i=1}^{k}n_{i}In\sigma_{i}\right)-\\ \sum_{i=1}^{k}n_{i}InK-B\sum_{i=1}^{k}n_{i}In\sigma_{i}-\sum_{i=1}^{k}\sum_{j=1}^{n_{i}}\left(\frac{N_{ij}}{K\sigma_{i}^{-B}}\right) \end{array}$$

According to the great likelihood estimation, the system of nonlinear equations for the estimated parameters *B*, *χ*, and *K* is listed, and the derivatives of *B*, *χ*, and *K* are, respectively, derived and set the derivatives to 0 to obtain the system of nonlinear equations, as shown in Equations (16), (17), and (18), respectively.

(16) $$\frac{\partial InL}{\partial B}=\left(\chi -1\right)\sum_{i=1}^{k}n_{i}In\sigma_{i}-\sum_{i=1}^{k}n_{i}In\sigma_{i}+\chi \sum_{i=1}^{k}\sum_{j=1}^{n_{i}}\left(\frac{N_{ij}}{K\sigma_{i}^{-B}}\right)In\sigma_{i}=0$$

(17) $$\begin{array}{l} \frac{\partial InL}{\partial \chi }=\left(\sum_{i=1}^{k}n_{i}\right)\frac{1}{\chi }+\left(\sum_{i=1}^{k}\sum_{j}^{n_{i}}InN_{ij}-\sum_{i=1}^{k}n_{i}InK+B\sum_{i=1}^{k}n_{i}In\sigma_{i}\right)-\\ {\sum_{i=1}^{k}\sum_{j=1}^{n_{i}}\left(\frac{N_{ij}}{K\sigma_{i}^{-B}}\right)}^{\chi }In\left(\frac{N_{ij}}{K\sigma_{i}^{-B}}\right)=0 \end{array}$$

(18) $$\frac{\partial InL}{\partial K}=-\left(\sum_{i=1}^{k}n_{i}\right)\frac{1}{K}-\left(\chi -1\right){\sum_{i=1}^{k}\frac{n_{i}}{K}+\chi \sum_{i=1}^{k}\sum_{j=1}^{n_{i}}\frac{1}{K}\left(\frac{N_{ij}}{K\sigma_{i}^{-B}}\right)}^{\chi }=0$$

#### 2.2.2. Parameter Optimization Algorithm (L-BFGS-B Algorithm)

To minimize the log-likelihood function for estimating the parameters *B*, *χ*, and *K* of the fatigue life model, the L-BFGS-B algorithm was adopted. L-BFGS-B is a bound-constrained quasi-Newton optimization method suitable for continuous nonlinear optimization problems. It approximates curvature information using limited memory and allows predefined lower and upper bounds to be imposed on each parameter during iteration. In the present fatigue model, these parameters have clear mathematical and physical admissible ranges. Specifically, the scale-related and Weibull distribution parameters must remain positive to ensure valid fatigue-life prediction and probability distribution. Therefore, box constraints were imposed to prevent nonphysical parameter estimates, such as negative scale parameters, negative Weibull shape parameters, or unrealistic divergence of the fatigue-life curve. These constraints improved the numerical stability of the calibration process and ensured that the optimized parameters retained physical interpretability. The BFGS algorithm is a quasi-Newton method that solves the optimization problem by iteratively updating an approximate Hessian matrix, which is computed as shown in Equation (19).

(19) $$B_{k+1}=B_{k}+\frac{y_{k}y_{k}^{T}}{y_{k}^{T}s_{k}}-\frac{B_{k}s_{k}s_{k}^{T}B_{k}}{s_{k}^{T}B_{k}s_{k}}$$

where *B_k_* is the Hessian matrix approximation for the *k*th iteration, $s_{k}=x_{k+1}-x_{k}$ is the parameter variation, and $y_{k}=\nabla f\left(x_{k+1}\right)-\nabla f\left(x_{k}\right)$ is the gradient variation.

The L-BFGS algorithm approximates the Hessian matrix by retaining information from only the most recent updates, thereby eliminating the need to store the entire Hessian matrix and significantly reducing memory requirements. Specifically, the L-BFGS algorithm uses two sets of vectors to store historical information. These vectors include the parameter variations (*S*) and gradient variation (*Y*), as shown in Equations (21) and (22), respectively. The low-rank approximation of the Hessian matrix is then constructed using these two sets of vectors.

(20) $$S=\left\{s_{k-m},\cdots ,s_{k-1}\right\}$$

(21) $$Y=\left\{y_{k-m},\cdots ,y_{k-1}\right\}$$

It is crucial in the L-BFGS-B algorithm to efficiently update and store the Hessian matrix approximation. The update formula is expressed as follows:

(22) $$B_{k+1}^{-1}=\left(I-\rho_{k}s_{k}y_{k}^{T}\right)B_{k}^{-1}\left(I-\rho_{k}y_{k}s_{k}^{T}\right)+\rho_{k}s_{k}s_{k}^{T}$$

where $B_{k}^{-1}$ is the inverse matrix of the approximate Hessian matrix at the *k*th iteration, $\rho_{k}$ is the scaling factor, $\rho_{k}=1/y_{k}^{T}s_{k}$.

Moreover, the L-BFGS-B algorithm is equipped to handle both unconstrained optimization problems and those with boundary constraints. During each iteration, a projection operation is required for each *x* to ensure that boundary constraints are satisfied. The specific boundary processing formula is shown in Equation (23).

(23) $$x_{i}=\min(\max(x_{i},l_{i}),u_{i})$$

where *x_i_* is the *i*th variable and *l_i_* and *u_i_* are the lower and upper bounds of the *i*th variable, respectively.

**Figure 3:**
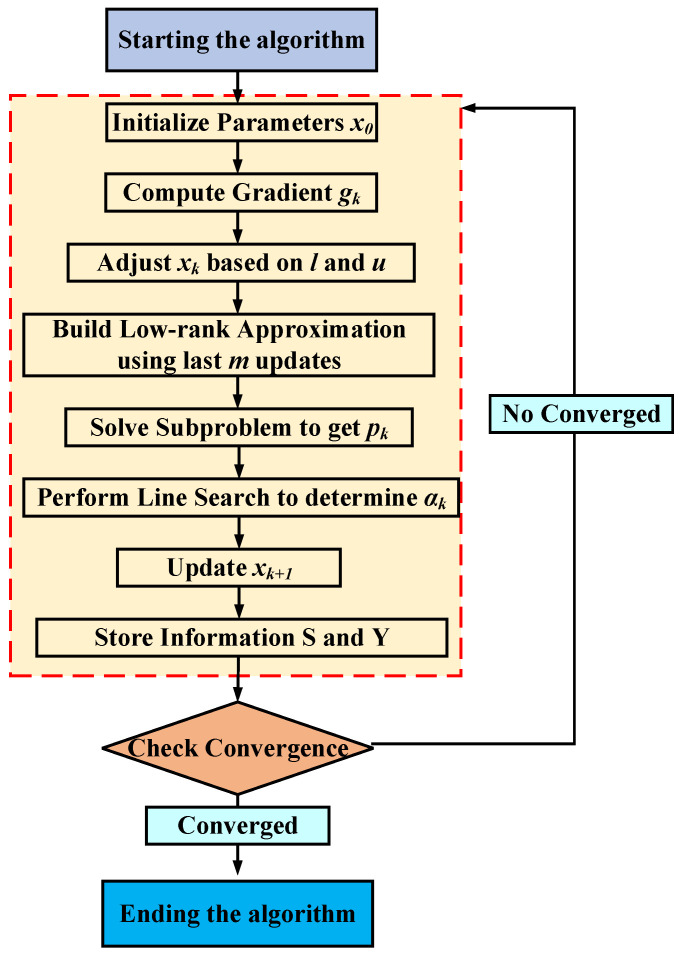
L-GFBS-B optimization algorithm flow.

The specific flow of the L-GFBS-B optimization algorithm is shown in Figure 3.

## 3. Composite Specimen Interface Fatigue Test and Analysis

### 3.1. Interface Fatigue Test

To investigate the fatigue performance of the track slab-CA mortar interface of CRTS II slab ballastless track, a series of interface fatigue tests will be carried out in this section. Prior studies have conducted interface tensile and push-out static load tests, determining the ultimate bearing capacity of the interface in both normal and tangential directions. The production of specimen materials and their dimensions have been detailed in the literature [2] and will not be reiterated here. This paper provides a detailed description of the loading and testing methods for the interface push-out and positive tensile fatigue tests, as well as the fatigue life of the interface under varying stress ratios.

**Figure 4:**
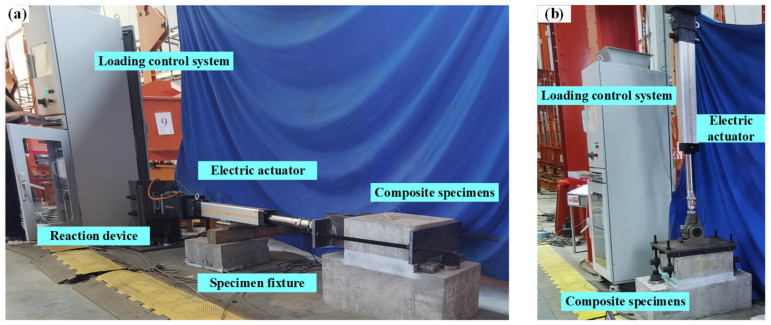
CRTS II slab ballastless track interface fatigue test setup ((**a**): push-out test; (**b**): positive tensile test).

**Figure 5:**
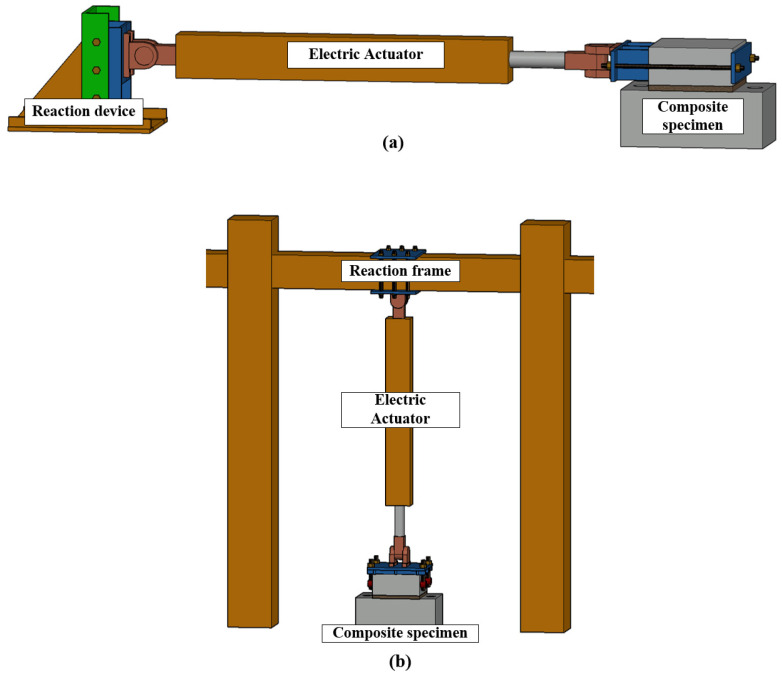
Schematic diagram of the interfacial fatigue test setup for the CRTS II slab ballastless track ((**a**): push-out test; (**b**): positive tensile test).

The fatigue loading system for the interface push-out and positive tensile fatigue tests adopted the SQ-ETH-50 electronic actuator and its associated loading control system, provided by Beijing Sizi Zhixin Science and Technology Co., Ltd. (Beijing, China). The electronic actuator had a maximum loading capacity of 50 kN, which was confirmed to meet the test requirements during preliminary pre-tests. The fatigue test setup primarily consisted of a custom-designed counterforce device, an electronic actuator, and a horizontal fixture for the test specimen. Initially, the custom counterforce device and composite specimen were anchored to the ground using anchor rods. The fixed end of the electronic actuator was then secured to the bearing surface of the counterforce device via bolts through the device’s pre-drilled holes. Subsequently, the specimen fixture was attached to the push-out specimen using the fixture connecting device, and the loading end of the electronic actuator was pre-anchored to the specimen fixture with bolts. Finally, the composite specimen was subjected to a horizontal uniform load and pre-anchored using a horizontal loading device. A 3D laser was employed for precise leveling and anchoring, as illustrated in Figure 4a and Figure 5a.

The positive tensile fatigue test setup primarily consisted of a counter-frame, a prefabricated counter-frame connection device, an electronic actuator, and a vertical fixture for the composite specimen. First, bolts were used to secure the prefabricated counterforce frame connection device to the counterforce frame, and the fixed end of the electronic actuator was attached to this connection device. Next, anchors were used to secure the composite specimen to the ground. Bolts were then used to attach the loading end of the electronic actuator to the specimen connection device. Finally, the specimen was subjected to a vertically upward uniform load at the interface, applied by the electronic actuator, and both the composite specimen and the electronic actuator were calibrated using a three-dimensional laser, as shown in Figure 4b and Figure 5b.

Studies have indicated that fatigue loading frequencies between 3 Hz and 16 Hz have minimal impact on the fatigue life of structures [40]. Accordingly, a frequency of 10 Hz was selected for this study, using a force control mode with a sinusoidal waveform.

The corresponding interfacial fatigue test results are shown in Table 1.

### 3.2. Fatigue Life Analysis of Interfaces

Based on the fatigue life model established in this study, the parameter estimates for the tangential and normal fatigue life models were derived using the interface fatigue test data and the previously described parameter estimation method, as presented in Table 2. It should be noted that the fatigue tests in this study were conducted under controlled dry and isothermal laboratory conditions, which enabled a systematic investigation of the mechanical fatigue behavior of the slab–CA mortar interface while minimizing the influence of external environmental variability. Accordingly, the proposed probabilistic fatigue model was established based on the experimentally observed mechanical degradation characteristics under the tested conditions. Although environmental factors such as temperature fluctuations, moisture ingress, freeze–thaw cycles, and hydrolysis were not explicitly incorporated into the current formulation, the model provides a fundamental framework for quantifying fatigue damage evolution and reliability degradation of the interface. Considering that these environmental actions may interact with cyclic loading in actual CRTS II ballastless tracks, future studies may further enhance the model by introducing environment-dependent parameters or correction factors, thereby extending its applicability to long-term durability assessment under more complex service conditions.

**Figure 6:**
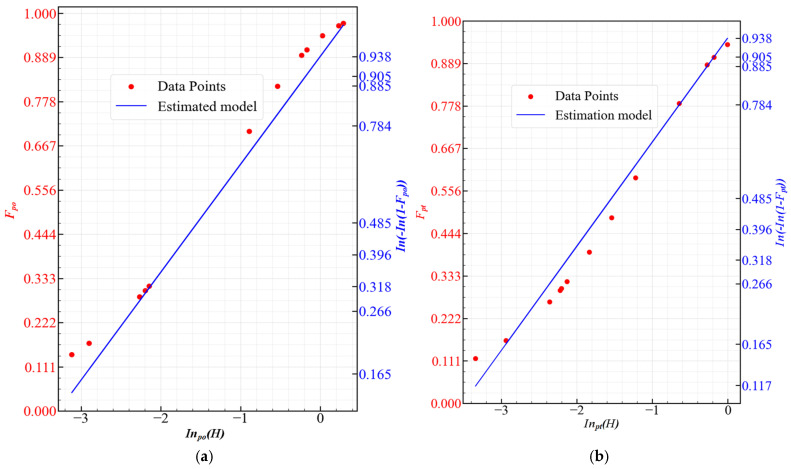
Weibull probability plot for the intermediate variable *H*, (**a**) push-out test, (**b**) positive tensile test.

In addition, by taking logarithms on both sides of Equation (10) and substituting the estimated parameters into the equation, the intermediate variables *In_po_*(*H*) and *In_ps_*(*H*) corresponding to the interface push-out test and the interface positive tensile test, respectively, can be obtained, as shown in Equations (24) and (25). The Weibull probability plot of the intermediate variable H is shown in Figure 6.

(24) $$In_{po}\left(H\right)=In\left(3.684\right)+\frac{1}{0.753}In\left(-In\left(1-F_{po}\right)\right)$$

(25) $$In_{pt}\left(H\right)=In\left(0.332\right)+\frac{1}{0.933}In\left(-In\left(1-F_{pt}\right)\right)$$

where *F_po_* and *F_pt_* are the Weibull distribution functions of the push-out test and the positive tensile test, respectively.

As shown in Figure 6, the empirical cumulative probabilities of each data point for the intermediate variable *H* exhibit linearity in the Weibull probability plot and closely align with the estimated model. The two-parameter Weibull distribution of the intermediate variable *H* was tested using the Kolmogorov–Smirnov (KS) test at the 95% significance level. These analyses indicate that the interface fatigue test data points corresponding to different stress ratios are well-described by the fatigue model proposed in this study.

#### 3.2.1. Analysis of PSN Curves

**Figure 7:**
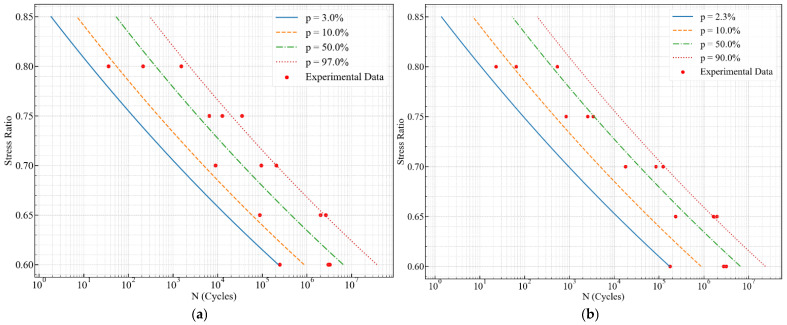
Interface fatigue PSN curve, (**a**) push-out test, (**b**) positive tensile test.

To further explore the fatigue-life prediction of the interface under varying stress ratios and failure probabilities, the P-S-N curves derived from Equation (1) were plotted for the interface push-out fatigue test and positive tensile fatigue test, as shown in Figure 7.

Figure 7a illustrates the tangential fatigue behavior of the interface between the track slab and CA mortar across different failure probabilities. The stress ratios ranged from 0.60 to 0.85, and the interface’s capacity to withstand stress decreased as the number of cycles increased. The curves, from left to right, corresponded to failure probabilities of 3%, 10%, 50%, and 97%, respectively. The experimental data were primarily concentrated between the 10% and 50% probability curves, indicating that most data points corresponded to failure probabilities within this range. A few data points were near the 97% probability curve, suggesting that the interface was more prone to failure at higher stress ratios in these instances. The probability of fatigue failure under tangential loading increased with the number of cycles, particularly in the high-stress ratio region. The data suggested that most interface failures occurred within the higher probability range, indicating that special attention should be given to fatigue at high stress ratios during design.

Figure 7b depicts the characteristics of the interface’s normal fatigue PSN curves. The stress ratios once again ranged from 0.60 to 0.85, with the probability curves corresponding to 2.3%, 10%, 50%, and 90%, respectively. These curves similarly demonstrated a gradual decrease in stress ratio as the number of cycles increased. The red data points represented the actual experimental data, which were more dispersed, with some points near the 50% probability curve and others closer to the 90% probability curve, indicating a broader distribution of interfacial fatigue failures under normal loading. Under normal loading, the probability of interface failure showed greater variability with stress ratio and number of cycles, and the failure data were more scattered. This indicated that normal fatigue behavior was more complex and might be influenced by various factors, such as material inhomogeneity or interfacial bond quality.

In summary, the failure probability of tangential fatigue at the interface exhibited a wider range, with a higher likelihood of failure, particularly in the high-stress ratio region. Conversely, the failure probability of normal fatigue was more concentrated, primarily distributed in the low to medium probability range. This may be due to the fact that, under normal loading, interface failure is predominantly governed by bond strength, resulting in a more consistent failure mode. Despite the similar range of stress ratios in the two plots, tangential and normal fatigue behaviors differed significantly. The interface was more prone to failure at high-stress ratios under tangential stresses, while it failed across a broader range of stress ratios under normal stresses, reflecting the distinct effects of these two loading modes on the interface’s fatigue performance. This difference may be attributed to the interface’s greater susceptibility to slip or spalling under tangential loading, leading to a higher probability of failure. Additionally, the material properties and bond quality of the interface between the track slab and CA mortar are more significantly affected under normal loading, which may contribute to the greater uncertainty observed in normal fatigue behavior.

To better understand the influence of the key input feature on the predicted fatigue life, we employ Partial Dependence Plots (PDPs). PDPs provide a clear visualization of how the model output varies with the input, quantitatively illustrating the relationship between stress ratio and predicted fatigue life.

In our probabilistic fatigue life model, the stress ratio (σ) is identified as the primary input feature, as it governs the interface loading conditions in both Push-out and Positive Tensile tests. The stress ratio determines the magnitude of cyclic loading applied to the slab–CA mortar interface, and consequently, strongly influences fatigue damage accumulation and predicted life.

**Figure 8:**
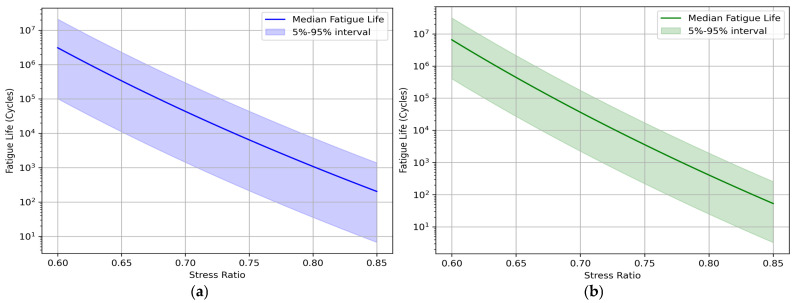
PDPs of interface fatigue life for CRTS II slab–CA mortar interface, (**a**) push-out test, (**b**) positive tensile test.

Using the calibrated multi-parameter Weibull model, we computed the median predicted fatigue life and the 5–95% probability interval for a range of stress ratios (0.6–0.85). The resulting PDPs are shown in Figure 8. The solid line represents the median predicted fatigue life, while the shaded area indicates the 5–95% probability interval. The plots clearly demonstrate that an increase in stress ratio results in a systematic decrease in predicted fatigue life, highlighting the sensitivity of interface fatigue behavior to this key feature.

The PDPs not only illustrate the monotonic relationship between stress ratio and predicted fatigue life but also emphasize the variability of predictions at each stress level. For the Push-out test (Figure 8a), the decrease is more pronounced at higher stress ratios, consistent with experimental observations of rapid interface failure. In contrast, the Positive Tensile test (Figure 8b) shows a comparatively wider 5–95% interval at intermediate stress ratios, indicating greater variability due to interface heterogeneity. These visualizations enhance the interpretability of the model and provide clear evidence of the feature–output relationship, directly addressing the reviewer’s comments on feature combination and dependence.

#### 3.2.2. Fatigue Life Probability Distribution Analysis

**Figure 9:**
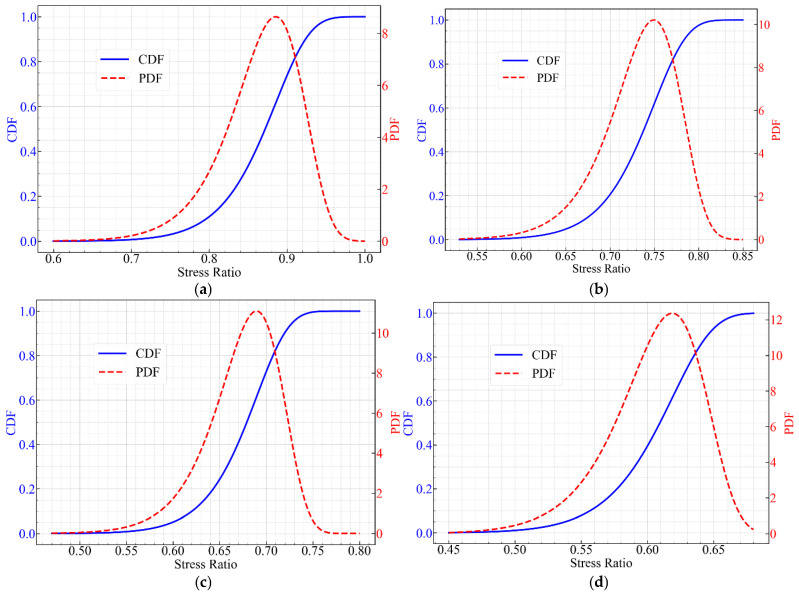
Interface tangential probability density function and cumulative distribution function, (**a**) N = 100, (**b**) N = 10,000, (**c**) N = 100,000, (**d**) N = 2,000,000.

**Figure 10:**
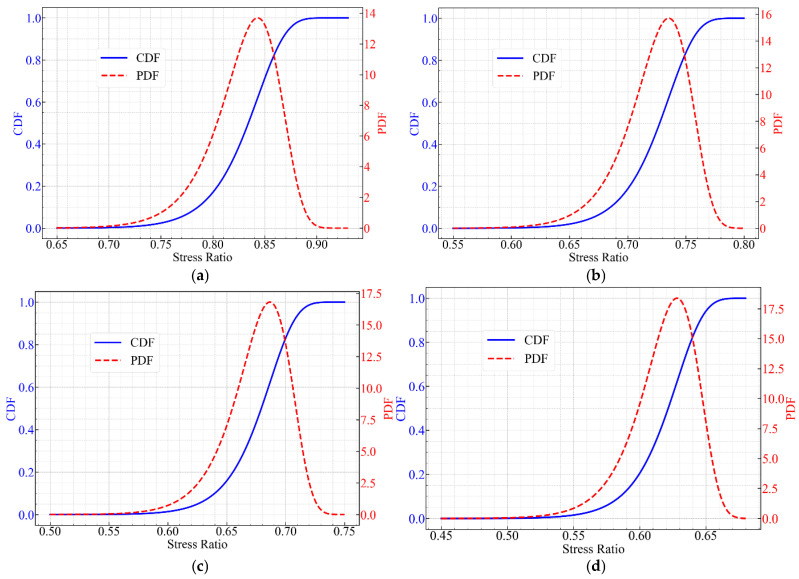
Interface vertical probability density function and cumulative distribution function, (**a**) N = 100, (**b**) N = 10,000, (**c**) N = 100,000, (**d**) N = 2,000,000.

To comprehensively assess structural safety and reliability, the distribution of stress ratios at different cycle counts was analyzed to investigate the concentration of stress ratios in specific regions and the cumulative probability. The probability density function (PDF) and cumulative distribution function (CDF) in the tangential and vertical directions of the interface are depicted in Figure 9 and Figure 10, respectively.

As shown in Figure 9, when the interface fatigue life was 100 cycles, the PDF peaked in the higher loading stress ratio region (around 0.9), and the CDF rose rapidly within this interval.

At high loading stress ratios, the specimen was subjected to stresses close to its fatigue limit in each fatigue cycle. High stress-ratio fatigue loading led to rapid damage accumulation within the material, increased the rate of crack propagation, and caused the specimen to quickly reach its fatigue life limit. When the interfacial fatigue life was 10,000 and 100,000 cycles, respectively, the PDF peak shifted to the lower stress ratio region (around 0.75 and 0.65), and the CDF curve became flatter accordingly. As the loading stress ratio decreased, the stress level in each cycle decreased, the crack propagation rate slowed, and the fatigue life was significantly extended. These specimens experienced loading stress ratios insufficient to rapidly initiate failure; however, fatigue damage continued to accumulate gradually, ultimately leading to failure. When the interfacial fatigue life reached 2,000,000 cycles, the PDF peak was located in the low loading amplitude region (around 0.6), and the CDF curve exhibited a much gentler slope, indicating a wider range of distribution. At very low loading stress ratios, the fatigue life of the specimens was significantly prolonged, demonstrating extremely strong fatigue resistance. At this stage, crack propagation within the material was very slow, damage accumulation remained nearly stable, and the material could withstand a large number of fatigue cycles. This behavior can be attributed to the influence of different loading stress ratios on stress concentration and grain slip behavior at the grain boundaries. At high loading amplitudes, slip system activity within the grain intensified, leading to easier initiation and propagation of fatigue cracks at the grain boundaries. Consequently, specimens subjected to high loading stress ratios typically exhibited shorter fatigue lives. Additionally, internal defects (e.g., micropores, inclusions) were more likely to serve as initiation points for cracks at high loading stress ratios, thereby accelerating the fatigue failure process. At low loading stress ratios, the effects of these defects were greatly minimized, thereby extending the material’s fatigue life.

As shown in Figure 10, for the specimen with a fatigue life of 100 cycles, the peak of the PDF curve was concentrated in the higher stress ratio region, around 0.85.

The CDF curve indicated that the cumulative probability rapidly increased to nearly 1.0 around 0.85, suggesting that most of the stress ratios in this specimen under high-stress ratio fatigue loading were concentrated in the higher range. This suggests that the specimen’s fatigue life was short at high loading stress ratios, with the vertical fatigue loading applied at the interface leading to rapid failure. For the specimen with a fatigue life of 10,000 cycles, the peak of the PDF shifted slightly leftward to around 0.75, indicating a decrease in the stress ratio. The relatively gentle upward trend of the CDF curve reflected a broader distribution of stress ratios compared to the previous specimen. This suggested that the specimen could withstand more fatigue loading cycles, but its lifespan was still relatively limited due to the high-stress ratio. For the specimen with a fatigue life of 100,000 cycles, the PDF peak continued to shift leftward to approximately 0.70, indicating a further decrease in the stress ratio. The CDF curve gradually increased over a wider range of stress ratios, indicating that the specimen had a longer fatigue life at lower stress ratios, with slower fatigue damage accumulation and greater durability under these conditions. For the specimen with a fatigue life of 2,000,000 cycles, the PDF peak further decreased to around 0.65, indicating that the stress ratio was lower and more widely distributed. The CDF curve showed a gentler upward trend, indicating that the stress ratio was more uniformly distributed across different regions. The specimen demonstrated a very high fatigue life, making it suitable for long-term low-stress-ratio fatigue loading conditions. The impact of vertical fatigue loading on the interface contact region was particularly significant. At high loading amplitudes, the vertical stress concentration at the interface likely led to the rapid expansion of microcracks in localized areas, ultimately resulting in specimen failure. As the loading stress ratio decreased (e.g., in specimens with a life of 2,000,000 cycles), the stress concentration effect diminished, and fatigue damage at the interface accumulated primarily through the slow expansion of microcracks and slip mechanisms, thereby prolonging the specimen’s fatigue life.

In summary, the proposed multi-parameter Weibull fatigue model can analyze interface fatigue life and estimate the probability distribution of the corresponding stress ratios at specified fatigue life values. Comparative analysis reveals clear differences in the fatigue behavior of the slab-CA mortar interface under tangential and vertical loading. Tangential loading is more prone to inducing stress concentration and rapid interface failure. Although the observed stress concentration may be partly affected by the boundary and edge effects of the push-out specimen, it also reflects an inherent shear-dominated damage mechanism of the slab-CA composite interface. Tangential loading generates interfacial shear stress and relative slip between the track slab and the CA mortar layer. Once local debonding or microcracking initiates, load transfer becomes localized, resulting in progressive stress concentration and accelerated interface failure. In real CRTS II ballastless tracks, similar degradation may be triggered by braking forces, rail creep, temperature-induced longitudinal deformation, and lateral train-track interaction.

By contrast, vertical loading shows better fatigue resistance at low loading amplitudes because the contact pressure is relatively uniformly distributed. However, this advantage weakens at high loading amplitudes. With continued cyclic loading, accumulated microcracks coalesce into dominant cracks, leading to a rapid loss of load-bearing capacity. As a result, the failure process changes from stable fatigue damage accumulation to accelerated crack propagation, and the final failure exhibits brittle fracture characteristics of the CA mortar. These findings provide guidance for optimizing the slab-CA mortar interface design. In practical applications, tangential fatigue degradation may be mitigated by controlling excessive longitudinal and lateral forces, improving interfacial bonding quality, reducing construction defects, optimizing restraint conditions, and strengthening monitoring of interface debonding or displacement. Future studies combining multiscale modeling and experimental methods are needed to further clarify the microscopic damage mechanisms and improve interface fatigue resistance through material and structural optimization.

From the viewpoint of model applicability, it should be noted that the proposed model is a reliability-oriented probabilistic fatigue-life model. It mainly captures the macroscopic statistical outcome of interfacial fatigue degradation based on the observed fatigue test data, rather than explicitly resolving the complete fracture-damage evolution process. Although interfacial debonding, microcrack coalescence, frictional slip, and local stress redistribution may contribute to the observed fatigue behavior, these mechanisms are not directly embedded in the current model formulation.

The probabilistic parameters calibrated in this study were obtained from specimens whose vertical dimension was kept consistent with that of the actual CRTS II track structure, whereas the longitudinal and transverse dimensions were reduced for laboratory testing. Therefore, the obtained parameters represent specimen-scale fatigue characteristics under a realistic vertical structural configuration. For application to a continuous track slab several meters long, further scale correction is required by considering the effective stressed interface area, critical interface length, spatial stress distribution, and full-scale monitoring data.

In addition, although the present PDF/CDF analysis characterizes the probabilistic distribution of interface fatigue life, microstructural examination after fatigue cycling would be valuable for revealing internal defects, microcrack initiation, crack coalescence, and interface deterioration. Future work should therefore combine probabilistic fatigue modeling with microstructural characterization, digital image correlation, cohesive fracture modeling, or multiscale simulation to strengthen the physical interpretation and field-scale predictive capability of interface fatigue degradation.

## 4. Conclusions

This paper derives the multi-parameter Weibull model for interface fatigue life, proposes the maximum likelihood method and the L-BFGS-B algorithm for optimizing parameter estimation, and conducts interface positive tensile and push-out tests. The theoretical model characterizing interface fatigue life is systematically established using test data, leading to the following key conclusions:

1. The analysis of interface fatigue test results from composite specimens of CRTS II slab ballastless track indicates that the proposed fatigue life probability model serves as a novel mathematical model for fatigue performance research.
2. The proposed multi-parameter Weibull model for fatigue life probability can be simplified to a two-parameter Weibull distribution for fatigue life at a specified stress ratio. Similarly, the corresponding stress ratio at a specified fatigue life can be simplified to a three-parameter Weibull distribution, enabling the determination of the probability distribution of stress ratios at a specified fatigue life.
3. The proposed fatigue model utilizes all available fatigue test data for parameter estimation, ensuring that the data across different stress ratios conform to the model. This approach minimizes statistical errors associated with limited test data and enhances the accuracy of parameter estimation.
4. Tangential loading tends to cause stress concentration and rapid failure at the interface, whereas vertical loading exhibits better fatigue life at low-stress ratios due to the uniform distribution of contact pressure.

From an engineering perspective, the proposed probabilistic fatigue model may support life-cycle assessment and maintenance planning of CRTS II slab ballastless tracks by estimating interface fatigue life and failure probability under specified stress ratios. Nevertheless, its field application still requires further validation using full-scale tests, structural stress analysis, long-term monitoring data, random loading spectra, and environmental correction factors. Future work should also integrate microstructural characterization, digital image correlation, cohesive fracture modeling, or multiscale simulation to strengthen the mechanistic interpretation and field-scale predictive capability of the model. The proposed modeling strategy may also be extended to other layered structures or composite interfaces governed by interfacial fatigue degradation.

## Figures and Tables

**Table 1:** Fatigue test results of track slab-CA mortar interface.

Stress Ratios	Push-Out			Positive Tensile		
	Specimen 1	Specimen 2	Specimen 3	Specimen 1	Specimen 2	Specimen 3
0.6	3,251,257	3,153,785	2,753,261	175,635	3,032,546	246,874
0.65	2,634,512	1,965,487	1,642,356	234,521	2,025,648	87,565
0.7	205,148	123,849	85,621	17,952	95,426	8964
0.75	35,124	3425	2546	846	12,658	6542
0.8	1536	536	65	23	213	36

**Table 2:** Parameter estimates for tangential and normal fatigue life models of interfaces.

Loading Method	B	*χ*	*K*
Push-out	27.64	0.763	3.684
Positive tensile	33.65	0.933	0.332

## Data Availability

The original contributions presented in this study are included in the article. Further inquiries can be directed to the corresponding authors.
